# Sinus Irrigation with N-Acetylcysteine after Endoscopic Sinus Surgery for Chronic Rhinosinusitis: A Preliminary Report of a Single-Blind Randomized Controlled Trial

**DOI:** 10.3390/diagnostics14151678

**Published:** 2024-08-02

**Authors:** Jee Hye Wee, Joo Hyun Park, Min Woo Park, Young Seok Choi, Hahn Jin Jung

**Affiliations:** 1 Department of Otorhinolaryngology-Head & Neck Surgery, Hallym University Sacred Heart Hospital, Hallym University College of Medicine, Anyang 14068, Republic of Korea; weejh07@gmail.com; 2 Department of Otorhinolaryngology-Head and Neck Surgery, Dongguk University Ilsan Hospital, Dongguk University College of Medicine, Goyang 10326, Republic of Korea; parkzzu19@naver.com; 3 Department of Otorhinolaryngology-Head and Neck Surgery, Kangdong Sacred Heart Hospital, Seoul 05355, Republic of Korea; subintern@naver.com; 4 Department of Otorhinolaryngology-Head and Neck Surgery, Chungbuk National University Hospital, Chungbuk National University College of Medicine, Cheongju 28644, Republic of Korea; yschoi@chungbuk.ac.kr

**Keywords:** chronic rhinosinusitis, endoscopic sinus surgery, N-acetylcysteine, biofilms, nasal lavage, sino-nasal outcome test

## Abstract

Nasal irrigation is crucial following endoscopic sinus surgery (ESS), especially for managing chronic rhinosinusitis (CRS). This study assessed the effectiveness of N-acetylcysteine (NAC) irrigation during the post-ESS period of patients with CRS without nasal polyposis. In this prospective, single-blind randomized controlled trial, 49 patients (NAC, *n* = 24; saline, *n* = 25) undergoing ESS were assigned to receive either NAC or saline irrigations twice daily for a month. The preoperative and postoperative assessments conducted included Lund–Macka (LM) and Lund–Kennedy (LK) endoscopic scores, the Nasal Obstruction Symptom Evaluation (NOSE) scale, and the Sino-Nasal Outcome Test-20 (SNOT-20). At 2 weeks, 1 month, and 3 months after the operation, endoscopic findings and symptoms were evaluated. Both groups showed no differences in age, sex, LM and LK scores, NOSE scale, and SNOT-20 preoperatively. In terms of the endoscopic findings regarding the sinonasal mucosa after ESS, the NAC group had slightly lower scores 2 weeks, 1 month, and 3 months after the operation, but this difference was not statistically significant. The NAC group showed significant improvement in VAS scores, namely, postnasal drip (1.0, *p* = 0.041), smell dysfunction (0.8, *p* = 0.003), and crust (1.5, *p* = 0.034), compared to the control group’s scores of 2.6, 4.7, and 3.6, respectively, 2 weeks after the operation, although no significant differences were observed in VAS scores for any symptoms 1 and 3 months after the operation. NAC was well tolerated, and no adverse events were reported. NAC irrigation showed benefits over saline irrigation in terms of improving postnasal drip, smell dysfunction, and crust after ESS for CRS without nasal polyposis in the immediate postoperative period.

## 1. Introduction

Chronic rhinosinusitis (CRS) is one of the most prevalent chronic diseases globally, with an estimated prevalence ranging from 1 to 19% [1]. Recent data from the Korea National Health and Nutrition Examination Survey (KNHANES) indicate a significant increase in CRS prevalence in Korea, rising from 1.84% (1.70–1.97) between 1998 and 2005 to 3.70% (3.18–4.23) by 2021 [2]. CRS involves recurring and persistent inflammation of the sinonasal tissues that is known not only to trigger significant physical symptoms but also negatively impact quality of life and substantially impair daily functioning [3]. In cases of CRS unresponsive to various medical therapies, endoscopic sinus surgery (ESS) remains the treatment of choice, aimed at alleviating these debilitating symptoms.

Nasal irrigation has long been a critical component of postoperative care for patients undergoing ESS [4]. The recent development of absorbable packing has significantly reduced patients’ discomfort following ESS [5]. Additionally, meticulous manipulation of endoscopic techniques and instruments has decreased the incidence of postoperative crust, synechia, and stenosis. However, evidence suggests that nasal saline irrigation helps alleviate postoperative symptoms by flushing the nasal cavity and sinuses, aiding in crust removal and the recovery of mucociliary function. Previous data have confirmed that nasal irrigation is safe, effective, and well tolerated by patients [6,7] and also shown that it leads to improved quality of life, symptom relief, fewer infections, and reduced antibiotic use [8,9].

The formulation of irrigation solutions has recently become a focal point of discussion. Beyond traditional saline solutions, various formulations, including hypertonic and hypotonic saline, mupirocin, xylitol, and Manuka honey, have been explored [10]. These diverse nasal irrigation formulas share the common goal of cleansing nasal passages and promoting mucosal healing, with several studies attesting to their efficacy [11].

Research has demonstrated that N-acetylcysteine (NAC), a precursor to the antioxidant glutathione, decreases biofilm formation [12,13], inhibits bacterial adherence [14], reduces the production of extracellular polysaccharide matrix [15], and destroys bacterial biofilms [12], although the corresponding mechanism has not been fully elucidated. Biofilms, which are microbial communities consisting of bacteria capable of self-replication on biological surfaces, cause persistent infections [16] and have been observed in many patients with CRS, adversely affecting treatment outcomes [17,18]. Bendouah et al. have shown a correlation between the biofilm-forming capacity of bacteria such as *Pseudomonas aeruginosa* and *Staphylococcus aureus* and poorer postsurgical outcomes [19,20]. While NAC has been widely used as a mucolytic agent via inhalation and oral and intravenous routes with a well-established safety profile [21,22], few studies have examined its effectiveness as an irrigation formula following ESS.

Consequently, this prospective randomized controlled study aims to determine whether NAC nasal irrigations are more effective than saline irrigation controls in improving post-ESS outcomes in CRS without nasal polyposis (CRSsNP) patients based on subjective and objective measures.

## 2. Materials and Methods

### 2.1. Participants

This prospective, single-blind (clinician only) randomized controlled study was conducted at Chungbuk National University Hospital. The Institutional Review Board of Chungbuk National University Hospital approved this study (IRB No. 2016-05-010), and informed consent was obtained from all participants. Due to the distinguishable appearances of NAC and saline and the necessity for subjects to mix the NAC with the saline, blinding of subjects was not feasible. This lack of blinding potentially introduced bias in symptom perception. To eliminate bias in the objective evaluation, blinding of the clinicians and investigators was prioritized.

Patients managed in a tertiary rhinology practice with CRSsNP requiring surgery were recruited and randomized between 1 March 2019 and 30 September 2019. Adult patients aged 18 or older undergoing ESS for CRSsNP were included. Exclusion criteria encompassed patients under 18 years, those with immunocompromised conditions or nasal polyps, and individuals who had taken steroids or antibiotics within three weeks prior to surgery.

### 2.2. Randomization and Intervention

Participants were randomized with respect to receiving nasal irrigation with either NAC (*n* = 24) or normal saline (*n* = 25). The NAC solution was prepared by adding two ampules of 800 mg/4 mL to 250 mL of normal saline. Each patient irrigated each nostril with 125 mL of the solution per side three times daily for 30 days. Surgeons were blinded to the participants’ group assignments. All patients received a standardized postoperative care regimen including a 7-day course of antibiotics (amoxicillin/clavulanate) and topical steroid spray (fluticasone furoate (220 micrograms) administered to each nasal cavity once daily). All ESS procedures were performed by the senior surgeon to maintain consistency in surgical care.

### 2.3. Preoperative Data Collection

Demographic and clinical data, including age, sex, medical and surgical history, and allergen sensitization, were recorded. All participants underwent the multiple-allergen simultaneous test (MAST) for 59 common aeroallergens. Allergen sensitization was defined as class 2 (≥0.7 IU/mL) or more for at least one allergen. Before undergoing ESS, patients completed questionnaires to document the severity of their CRS. They graded their symptoms (nasal obstruction, rhinorrhea, postnasal drip, alteration in sense of smell, epistaxis, and crust) using the widely accepted and validated 10-point visual analog scoring system (VAS). Additionally, they assessed the impact of CRS on their quality of life using the Nasal Obstruction Symptom Evaluation (NOSE) scale and Sino-Nasal Outcome Test 20 (SNOT-20). The treating surgeon evaluated the sinonasal mucosa using the Lund–Kennedy (LK) scoring system. All patients underwent preoperative computed tomography (CT), which was assessed using the Lund–Mackay (LM) scoring system.

### 2.4. Postoperative Data Collection

Follow-up visits were conducted at 2 weeks, 1 month, and 3 months following the operation. At each visit, patients reassessed their symptoms, quality of life, and endoscopic scores using the same preoperative scales (i.e., VAS, NOSE scale, SNOT-20, and LK endoscopic score). Two investigators assessed postoperative endoscopic findings of nasal mucosa for synechia, edema, pus discharge, granulation, stenosis, and crust using the same grading system on a scale of 0 to 2 or 3 [23]. The two investigators were blinded to the preoperative state, allocated group of the patients, and time interval of the follow-up. Furthermore, open-ended surveys were utilized to evaluate patient compliance, tolerance, and the side effects of the irrigation solution.

### 2.5. Statistical Analysis

Descriptive statistics and distribution were analyzed for baseline characteristics. Continuous variables are expressed as the mean ± standard deviation (SD), and categorical variables are expressed as proportions. Chi-square test for binary variables and Mann–Whitney U test for continuous variables were performed to check for imbalances in descriptive characteristics between the groups. Paired t-tests were used to compare the LK endoscopic score, NOSE scale, SNOT-20 value, and VAS score before and after ESS. Unpaired t-tests were used to examine the differences in NAC and control groups. A repeated-measures analysis of variance (ANOVA) was used to assess changes in variables between two groups during the postoperative follow-up period. Statistical significance was set at *p* < 0.05. All analyses were performed using SPSS 26.0 (IBM, Armonk, NY, USA).

## 3. Results

### 3.1. Study Overview

**Figure 1:**
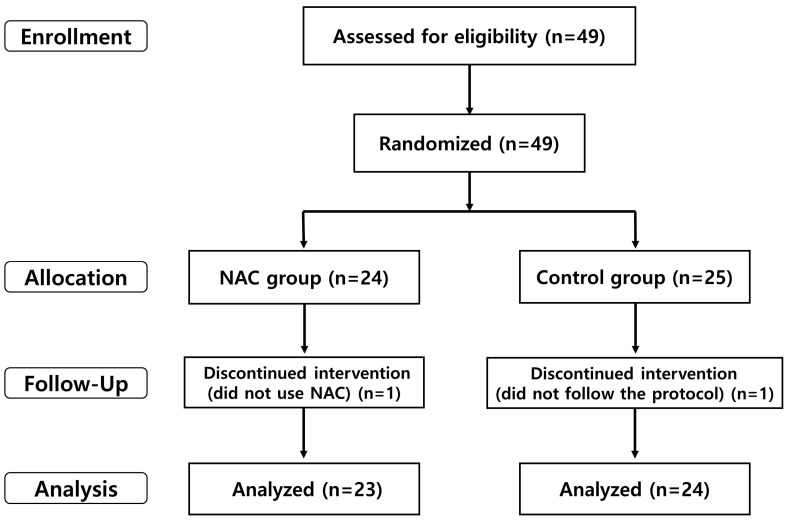
Participant flowchart. NAC = N-acetylcysteine.

Figure 1 illustrates the flow of participants throughout the study. A total of 49 patients were randomized. In the saline group (*n* = 25), one patient was excluded due to improper irrigation. In the NAC group (*n* = 24), one patient was excluded for using saline without adding NAC. Consequently, analyses were conducted on 24 patients who used saline irrigation and 23 who used NAC irrigation.

### 3.2. Descriptive Characteristics

The baseline demographic and clinical characteristics of the participants are presented in Table 1. Of the 47 patients eligible for this study, 25 were male (53.2%) and 22 were female (46.8%), with a mean age of 40.4 ± 3.5 years. There was no significant difference between two groups in terms of sex (*p* = 0.837), age (*p* = 0.112), or allergen sensitization (*p* = 0.845). Preoperative measures of sinusitis severity, both subjective (NOSE scale, *p* = 0.659; SNOT-20, *p* = 0.558) and objective (LM score, *p* = 0.500; LK score, *p* = 0.658), were similar across both groups.

### 3.3. Outcome Measures

**Figure 2:**
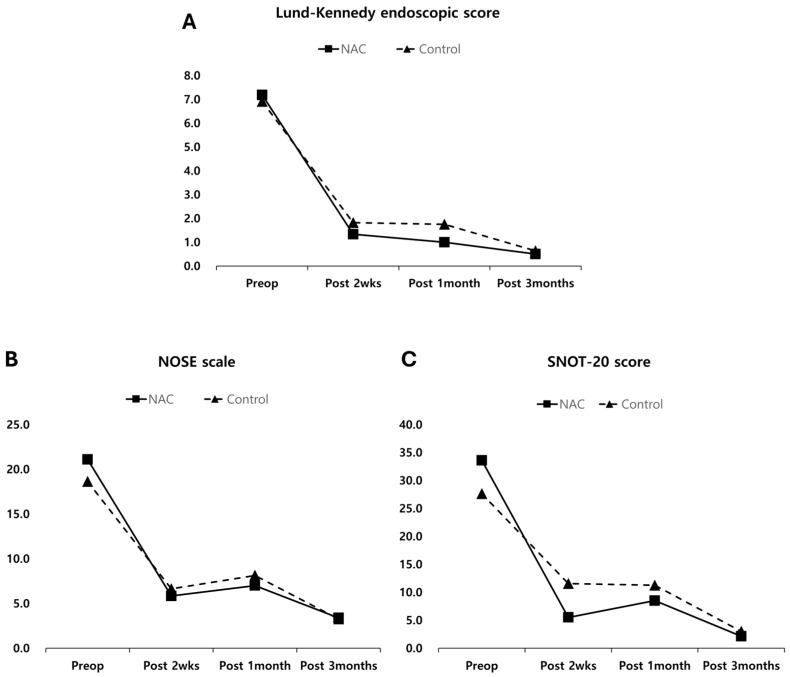
Objective and subjective assessments ((**A**): Lund-Kennedy endoscopic score, (**B**): NOSE scale, and (**C**): SNOT-20 score) before the operation and 2 weeks, 1 month, and 3 months after the operation. NAC = N-acetylcysteine.

**Figure 3:**
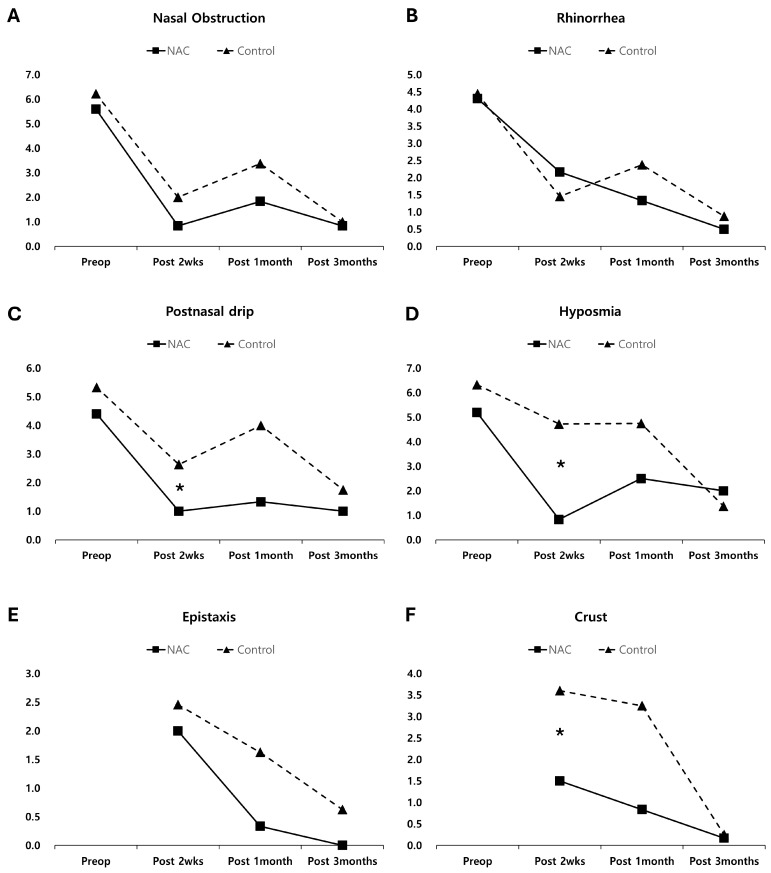
The VAS scores for individual symptoms ((**A**): nasal obstruction, (**B**): rhinorrhea, (**C**): postnasal drip, (**D**): hyposmia, (**E**): epistaxis, and (**F**): crust) before the operation and 2 weeks, 1 month, and 3 months after the operation (* *p* < 0.05 by unpaired t-tests between NAC and control groups). NAC = N-acetylcysteine.

In both groups, postoperative improvements were significant in terms of LK endoscopic score, NOSE scale, and SNOT-20 at 2 weeks, 1 month, and 3 months after ESS (all *p* < 0.05, Figure 2). Additionally, VAS score revealed significant reductions in symptoms of nasal obstruction, rhinorrhea, postnasal drip, and hyposmia at 3 months after ESS in both groups (all *p* < 0.05, Figure 3).

Comparative analyses between the NAC and control groups showed no significant differences in LK endoscopic scores at 2 weeks, 1 month, and 3 months after the operation (all *p* > 0.05, Figure 2A). Subjective assessments conducted via the NOSE scale and SNOT-20 also indicated no significant differences between the two groups (all *p* > 0.05, Figure 2B,C). However, at 2 weeks after the operation, the VAS scores for individual symptoms for postnasal drip (*p* = 0.041), hyposmia (*p* = 0.003), and crust (*p* = 0.034) were significantly lower in the NAC group compared to the control group (Figure 3). No significant differences were observed in VAS scores for any symptoms at 1 and 3 months after surgery (all *p* > 0.05).

Endoscopic findings regarding sinonasal mucosa after ESS revealed generally lower scores for synechia, edema, discharge, granulation, stenosis, and crust in the NAC group compared to the saline group, though these differences were not statistically significant (all *p* > 0.05, Table 2). Additionally, there was no significant difference in the improvement in endoscopic findings between the NAC and control groups (as assessed via repeated-measures ANOVA analyses, all *p* > 0.05).

### 3.4. Tolerability, Adverse Effects, and Compliance

Subjective tolerability and compliance were comparable between both groups. No serious adverse effects were reported in either group.

## 4. Discussion

This prospective, randomized trial assessed the efficacy of topical NAC irrigations compared with saline irrigations as postoperative treatments following ESS for patients with CRSsNP. Overall and final subjective symptoms, along with objective endoscopic scores, showed no significant differences between the two groups. However, postnasal drip, hyposmia, and crust appeared to improve in the short term in the NAC group compared to the saline irrigation group.

Biofilms, which are complex networks of pathogens encased within protective extracellular polymeric substances [24], play a pivotal role in the persistence of postoperative symptoms and ongoing mucosa inflammation and infections. These biofilms enhance bacterial resistance and survival, rendering standard chemical (antibiotics, disinfectants) and biological (viruses, protists) antimicrobial agents less effective [25]. *Staphylococcus aureus*, often identified as a prevalent biofilm-forming organism [26], has been linked to surgically recalcitrant diseases [27,28,29]. Traditional antibiotic therapies, despite being the conventional approaches to eradicate these biofilm, often fail due to poor penetration into the biofilm’s deeper layers [30]. Furthermore, a previous study showed that in post-surgical patients, only saline irrigation is not associated with a distinct sinonasal microbiota [31]. Various studies have explored different irrigation solutions. There have been studies suggesting benefits of hypertonic saline solutions, while others advocate for the effectiveness of xylitol. Among these, some focus on the potential breakdown of bacterial biofilms.

Inspired by the need for effective biofilm management, the present study utilized NAC, known for its role in controlling bacterial biofilm growth in human disease [16]. As a precursor to antioxidant glutathione, NAC helps maintain reactive oxygen species (ROS) homeostasis [32] and has been widely and safely used through inhalation and oral and intravenous routes [21,22]. Although its mechanisms are not fully understood, NAC has demonstrated potential in both inhibiting biofilm formation and disrupting established biofilms [16]. Indeed, several in vitro studies have reported that NAC decreases the formation of biofilms by a variety of bacteria [12,33,34,35]. Additionally, NAC may reduce the production of extracellular polysaccharide matrix [15] and promote the disruption of mature biofilms [36,37]. These effects are attributed to its mucolytic properties [38,39] and bacteriostatic behavior [40]. As a mucolytic agent, NAC reduces the viscosity of mucus, potentially aiding in clearing the sinuses and reducing postoperative crusting. This can lead to quicker recovery and less discomfort for patients postoperatively. Several studies have demonstrated that NAC not only decreases biofilm formation and inhibits bacterial adherence but also reduces the production of extracellular polysaccharide matrix and the cell viability of a variety of Gram-negative and Gram-positive bacteria [12,13,14,15,37,41]. Furthermore, NAC addresses the issue of antibiotic resistance by increasing the ability of antibiotics to permeate into the deepest layers of biofilm, thereby enhancing the efficacy of conventional drug therapy with antimicrobial agents [16]. NAC is generally safe and well tolerated even at high doses, offering a highly favorable risk/benefit ratio and a low rate of adverse events [38]. In this study, we explored these aspects and found that although NAC irrigation did not produce significant overall results, it did result in notable improvements in postnasal drip, hyposmia, and crust in the immediate postoperative period.

The VAS scores for postnasal drip and hyposmia showed significant improvement in the second postoperative week, but some deterioration was observed one month later, although there was no significant difference between the two groups. The increase in VAS scores at one month was thought to be due to the effects of edema. According to the endoscopic findings, edema scores increased from two weeks to one month after ESS. The scores for crusts, which had little correlation with edema and were primarily affected by irrigation, decreased continuously from two weeks to three months after ESS.

Contrary to expectations, irrigation using NAC showed no significant differences compared to the saline irrigation group. There are some explanations for the present findings. First, the transition from in vitro efficacy to clinical relevance was challenging to demonstrate. The environment within the sinus after ESS is complex, and biofilm properties can vary significantly, meaning that the effectiveness of NAC may not be applicable or noticeable in clinical outcomes. Second, NAC is known for its mucolytic effects, which help thin mucus, making it easier to clear it from the nasal cavity and sinuses. This property is beneficial in managing CRS symptoms. However, because the main concerns after ESS are mucosa healing and reducing inflammation rather than mucus clearance, the use of NAC irrigation may not have had a significant effect on postoperative wound healing. Third, the variability in patient responses to NAC could also be a factor. CRS is a heterogeneous disease with multiple underlying etiologies and patient-specific factors that could affect treatment outcomes. Additionally, the concentration and frequency of NAC used could have contributed to the lack of significant findings. Lastly, saline irrigation itself is quite effective in postoperative care as it helps to keep nasal passages clean and moist, reduce crusting, and aid mucosal healing. The baseline efficacy of saline might overshadow potential incremental improvements brought by NAC. In this study, all the participants showed improvement in subjective and objective measures after ESS, but if NAC and saline irrigation were compared by selecting patients who had poorer postoperative outcomes and were particularly likely to be affected by biofilms, significant differences might have been obtained.

Our study has several limitations. As previously mentioned, this study is the first study to investigate NAC irrigations; however, we were unable to assess the optimal dosage or frequency. Furthermore, blinding patients to the irrigation treatment was not feasible, as NAC differs from saline in appearance. This lack of blinding may have introduced bias into the self-reported symptom scores. Nevertheless, efforts to mitigate experimenter bias were made by ensuring that the surgeon and investigators assessing postoperative endoscopic findings were blinded to the type of irrigation used. Additionally, there is no consensus on the appropriate frequency or dosage for NAC sinus irrigations. The concentration used in our study was based on in vitro studies and tolerability data, although these data are variable. Another limitation is that we only evaluated the three-month postoperative period. This timeframe is restrictive because, in the immediate post-ESS period, the most crucial factor for continued improvement is likely the cleansing of nasal passages and the removal of crusts to prevent scarring. Therefore, regardless of the irrigation solution used, the primary focus is on its ability to facilitate this removal. This study was also constrained by its small sample size and short-term follow up. Future research should involve a larger cohort and extend the follow-up period to derive statistically significant differences. Additionally, the lack of biofilm assessment is another notable limitation of this study.

## 5. Conclusions

This preliminary study evaluated the efficacy of NAC versus saline irrigations in patients with CRSsNP who had undergone ESS. Despite the relatively small sample size, NAC irrigations did not statistically improve endoscopic findings or quality of life compared to the saline controls. However, there was a trend suggesting that NAC may improve PND, hyposmia, and crusting in the immediate postoperative period. Future studies should be more conservatively powered to detect smaller effect sizes, employ longer follow-up intervals to assess quality-of-life outcomes more accurately, explore cost issues affecting patient compliance, and evaluate the optimal frequency and dosing of NAC.

## Figures and Tables

**Table 1:** Baseline demographics of the participants.

Variables	Total (*n* = 47)	NAC Group (*n* = 23)	Control Group (*n* = 24)	*p*-Value
Age (years old, mean ± SD)	40.4 ± 3.5	34.5 ± 5.1	45.8 ± 4.5	0.112
Sex (*n*, %)				0.837
Male	25 (53.2)	13 (56.5)	12 (50.0)	
Female	22 (46.8)	10 (43.5)	12 (50.0)	
Allergen sensitization (*n*, %)				0.845
Yes	22 (46.8)	10 (43.5)	12 (50.0)	
No	25 (53.2)	13 (56.5)	12 (50.0)	
Lund–Mackay score (mean ± SD)	15.5 ± 4.8	16.3 ± 5.7	14.8 ± 4.1	0.500
Lund–Kennedy endoscopic score (mean ± SD)	7.0 ± 1.4	7.2 ± 1.4	6.9 ± 1.4	0.658
NOSE scale (mean ± SD)	20.0 ± 10.7	21.1 ± 8.5	18.6 ± 13.4	0.659
SNOT-20 (mean ± SD)	30.9 ± 20.7	33.6 ± 21.6	27.6 ± 20.6	0.558

NAC = n-acetylcysteine, SD = standard deviation, NOSE = Nasal Obstruction Symptom Evaluation, and SNOT = sino-nasal outcome test.

**Table 2:** Endoscopic finding scores at 2 weeks, 1, and 3 months after surgery.

Endoscopic Findings	POD 2 Weeks (Mean ± SD)	POD 1 Months (Mean ± SD)	POD 3 Months (Mean ± SD)	*p*-Value *
Synechia				0.723
NAC	0.00 ± 0.00	0.00 ± 0.00	0.00 ± 0.00	
Control	0.18 ± 0.18	0.25 ± 0.18	0.25 ± 0.25	
Edema				0.511
NAC	0.33 ± 0.21	0.50 ± 0.84	0.11 ± 0.11	
Control	0.45 ± 0.16	0.63 ± 0.26	0.25 ± 0.13	
Discharge				0.413
NAC	0.33 ± 0.21	0.17 ± 0.17	0.00 ± 0.00	
Control	0.27 ± 0.19	0.50 ± 0.27	0.08 ± 0.08	
Granulation				0.892
NAC	0.17 ± 0.17	0.00 ± 0.00	0.00 ± 0.00	
Control	0.18 ± 0.18	0.13 ± 0.12	0.13 ± 0.12	
Stenosis				1.000
NAC	0.00 ± 0.00	0.00 ± 0.00	0.00 ± 0.00	
Control	0.18 ± 0.18	0.13 ± 0.12	0.13 ± 0.12	
Crust				0.879
NAC	0.83 ± 0.17	0.33 ± 0.21	0.17 ± 0.16	
Control	0.67 ± 0.19	0.25 ± 0.16	0.25 ± 0.16	

* *p*-value for the repeated-measures analysis of variance. POD = postoperative day, SD = standard deviation, and NAC = n-acetylcysteine.

## Data Availability

Data are contained within the article.
